# Risk factors and survival prediction of young breast cancer patients with liver metastases: a population-based study

**DOI:** 10.3389/fendo.2023.1158759

**Published:** 2023-06-23

**Authors:** Chen-Chen Pu, Lei Yin, Jian-Ming Yan

**Affiliations:** ^1^ Department of Breast and Thyroid Surgery, The First People’s Hospital of Taicang, Taicang Affiliated Hospital of Soochow University, Taicang, Jiangsu, China; ^2^ Department of Breast and Thyroid Surgery, Wuzhong People’s Hospital of Suzhou City, Suzhou, Jiangsu, China

**Keywords:** young patient, breast cancer, liver metastases, SEER database, nomogram

## Abstract

**Background:**

The risk and prognosis of young breast cancer (YBC) with liver metastases (YBCLM) remain unclear. Thus, this study aimed to determine the risk and prognostic factors in these patients and construct predictive nomogram models.

**Methods:**

This population-based retrospective study was conducted using data of YBCLM patients from the Surveillance, Epidemiology, and End Results database between 2010 and 2019. Multivariate logistic and Cox regression analyses were used to identify independent risk and prognostic factors, which were used to construct the diagnostic and prognostic nomograms. The concordance index (C-index), calibration plot, receiver operating characteristic (ROC) curve, and decision curve analysis (DCA) were used to assess the performances of the established nomogram models. Propensity score matching (PSM) analysis was used to balance the baseline characteristics between the YBCLM patients and non-young patients with BCLM when comparing overall survival (OS) and cancer-specific survival (CSS).

**Results:**

A total of 18,275 YBC were identified, of whom 400 had LM. T stage, N stage, molecular subtypes, and bone, lung, and brain metastases were independent risk factors for LM developing in YBC. The established diagnostic nomogram showed that bone metastases contributed the most risk of LM developing, with a C-index of 0.895 (95% confidence interval 0.877–0.913) for this nomogram model. YBCLM had better survival than non-young patients with BCLM in unmatched and matched cohorts after propensity score matching analysis. The multivariate Cox analysis demonstrated that molecular subtypes, surgery and bone, lung, and brain metastases were independently associated with OS and CSS, chemotherapy was an independent prognostic factor for OS, and marital status and T stage were independent prognostic factors for CSS. The C-indices for the OS- and CSS-specific nomograms were 0.728 (0.69–0.766) and 0.74 (0.696–0.778), respectively. The ROC analysis indicated that these models had excellent discriminatory power. The calibration curve also showed that the observed results were consistent with the predicted results. DCA showed that the developed nomogram models would be effective in clinical practice.

**Conclusion:**

The present study determined the risk and prognostic factors of YBCLM and further developed nomograms that can be used to effectively identify high-risk patients and predict survival outcomes.

## Introduction

Breast cancer (BC) is the most frequently diagnosed malignancy among women worldwide and is also the main cause of cancer-related deaths in women (1). BC incidence increases with age, with the majority of initially diagnosed patients being ≥ 40 years. Age is also a vital factor for the survival of patients with BC; compared with older patients, young patients often have an inferior prognosis (2–4). The European Society for Medical Oncology guidelines define young patients with BC (YBC) as those aged < 40 years (5). BC in young patients is relatively rare, accounting for approximately 5–15% of all invasive BC cases (6, 7). However, some studies have revealed that BC in young patients is much more aggressive and correlates with a poor prognosis (8–10). All this evidence suggests differences in features and survival between YBC and older patients with BC (OBC).

Although the survival rate of patients with BC has significantly improved owing to recent advances in early diagnoses and comprehensive treatment strategies, the occurrence of distant metastasis cannot be ignored. An estimated 20–30% of patients with BC develop metastases during diagnosis or treatment, and metastases account for approximately 90% of cancer-related deaths (11). Distant metastases can severely disrupt the 5-year overall survival (OS) rate: BC patients with distant metastases have a 5-year OS rate of 25%, whereas those without distant metastases have a rate of 80% (12, 13). BC exhibits metastatic propensity in distinct organs—called metastatic heterogeneity—and results in varied treatment and survival responses. The common metastatic patterns of BC include the bone, lung, liver, and brain. Among these organs, the occurrence rate of liver metastases (LM) is lower than bone and lung metastases, but with an estimated 5-year OS rate of 8.5%, which is worse than that of bone and lung metastases (13). However, few large-scale population studies have focused on YBC with LM (YBCLM). Our understanding of the clinicopathological features and prognosis of these patients, especially the distinct differences between YBCLM and OBC with LM (OBCLM), is limited. To address this deficiency, we performed a retrospective population-based study using data from the Surveillance Epidemiology and End Results (SEER) database to 1) identify the risk factors for LM in YBC; 2) compare the features and survival between YBCLM and OBCLM; and 3) establish risk and prognostic nomograms for YBCLM to help clinicians accurately predict the development of LM and survival in YBC.

## Materials and methods

### Patients

Anonymized patient-level data were obtained from the SEER database, which is the largest publicly available database of patients with cancer in the United States and contains high-quality information on cancer incidence and survival for approximately 26% of the country’s population (14). The database (Incidence - SEER Research Plus Data, 17 registries, Nov 2021 Sub, 2000–2019) was searched to identify BC cases diagnosed between 2010 and 2019 using histology codes (ICD-0-3:8500) and their corresponding locations (Site recode ICD-O-3/World Health Organization [WHO] 2008: breast) (15). Three types of BC were identified: YBC without LM, YBCLM, and OBCLM. Patients who met the following criteria were included: 1) breast cancer as the only primary malignancy and 2) adequate information on survival time and follow-ups. Since identifying information of patients with malignancies was removed from the SEER database, ethics committee review and informed consent were not required.

### Variables

The following baseline demographic and clinicopathological characteristics of the included patients were obtained using the SEER*Stat 8.4.0 software: age at diagnosis (<30yr, ≥30yr), race (white, black, other), sex (female, male), marital status (married, single, Others), pathological differentiation (grade I, II, III, IV), TNM stage (TNM-I, II, III, IV), molecular subtype (Luminal A, Luminal B, HER2, Triple-negative), LM (yes, no), lung, bone, and brain metastases (yes, no), surgeries (no surgery, breast conserving surgery, mastectomy), radiotherapy (yes, no/unknown), and chemotherapy (yes, no/unknown). We chose OS and cancer-specific survival (CSS) as primary endpoints. OS was defined as the time between the initial diagnosis of BC and death from all causes or the last follow-up, whereas CSS was defined as the time between the initial diagnosis of BC and death due to BC or the last follow-up. All these data were required in this study; therefore, patients without these data were excluded.

### Statistical analysis

Continuous and categorical data are presented as mean ± standard deviation and numbers with percentages, respectively. Differences in continuous and categorical data were evaluated using t-tests and chi-square tests. To identify the independent risk factors for predicting LM in YBC, a multivariate logistic regression analysis was performed for odds ratios (ORs) with 95% confidence intervals (CIs). Independent risk factors were incorporated into the construction of the risk nomogram. The Kaplan–Meier method was used to assess OS and CSS, and the log-rank test was used to assess differences. A multivariate Cox regression analysis was performed to assess the influence of demographic and clinicopathological characteristics on OS and CSS as hazard ratios (HRs) with 95% CIs and to identify independent prognostic factors. Propensity score matching (PSM) analysis was widely used in observational study to reduce selection on bias across different groups (16), which was conducted to balance the baseline characteristics between the YBCLM and OBCLM groups when comparing the OS and CSS. OS- and CSS-specific nomograms of YBCLM were established on the basis of independent prognostic factors obtained from the multivariate Cox regression analysis to predict the survival of YBCLM. Harrel’s consistency index (C-index) was calculated the prediction performance of the nomogram models (17). The receiver operating characteristic (ROC) curve were used to validate the accuracy of the established nomogram, and a calibration curve plot was drawn to confirm the discrimination of the nomogram. A decision curve analysis (DCA) was conducted to determine the clinical utility of the established nomogram models by quantifying net income under different threshold probabilities (18). All statistical analyses were performed using R software (http://www.r-project.org). All P-values were two-sided, and P < 0.05 was regarded as statistically significant.

## Results

### Patients’ baseline characteristics

According to the inclusion criteria, the data of 18,275 YBC diagnosed between 2010 and 2019 were extracted from the SEER database. Among them, 400 patients had LM at the initial diagnosis. **Table 1** presents the baseline demographic and clinicopathological characteristics as well as the treatment of YBC with or without LM. Significant differences were detected between two groups in terms of age, race, marital status, pathological grade, molecular subtype, etc. YBCLM tended to be younger, with an average age of 33.6 (33.6 ± 4.2 vs 34.6 ± 3.8 years), non-White (33.5 vs 28.5%), and unmarried (46.0 vs 39.4%). Regarding molecular subtypes, YBC with luminal B (29.8 vs. 19.4%) and HER2 (21.0 vs. 7.0%) were most likely to develop LM. Grade III (65.7%) was the most common degree of pathological differentiation. The most common stages of T and N were T2 (35.2%) and N1 (54.8%). In terms of treatment, 34.5% of YBCLM underwent surgery, 91.5% received chemotherapy, and 29.7% received radiotherapy.

**Table 1:** The demographic and clinicopathological characteristics of YBC patients with and without liver metastases.

Characteristics	YBC patients with LM (N=400)	YBC patients without LM (N=17878)	P Value
Age (mean + SD)	33.6 ± 4.2	34.6 ± 3.8	P<0.01
Sex			
Female	399 (99.8%)	17831 (99.7%)	1.00
Male	1 (0.2%)	47 (0.3%)	
Race			
White	266 (66.5%)	12774 (71.5%)	P<0.01
Black	91 (22.8%)	2613 (14.6%)	
Other	43 (10.7%)	2491 (13.9%)	
Laterality			
Left	192	8973	P=0.65
Right	208	8901	
Bilateral	0	4	
Marital status			
Married	216 (54.0%)	10829 (60.6%)	P<0.01
Single	159 (39.8%)	5682 (32.7%)	
Others	25 (6.2%)	1367 (7.7%)	
Grade			
I	9 (2.3%)	1151 (6.4%)	P<0.01
II	125 (31.2%)	6039 (33.8%)	
III	263 (65.7%)	10625 (59.4%)	
IV	3 (0.8%)	63 (0.4)	
AJCC-T			
T0-1	39 (9.8%)	6678 (37.4%)	P<0.01
T2	145 (35.2%)	8320 (46.5%)	
T3	112 (28.0%)	2111 (11.8%)	
T4	104 (26.0%)	769 (4.3%)	
AJCC-N			
N0	68 (17.0%)	9006 (50.4%)	P<0.01
N1	219 (54.8%)	6596 (36.9%)	
N2	51 (12.7%)	1378 (7.7%)	
N3	62 (15.5%)	898 (5.0%)	
Subtype			
Luminal A	132 (33.0%)	9523 (53.3%)	P<0.01
Luminal B	119 (29.8%)	3464 (19.4%)	
HER2	84 (21.0%)	1250 (7.0%)	
Triple-negative	65 (16.2%)	3641 (20.3%)	
Surgery			
BCS	37 (9.3%)	6370 (35.6%)	P<0.01
Mastectomy	101 (25.2%)	10385 (58.1%)	
No surgery	262 (65.5%)	1123 (6.3%)	
Radiation			P<0.01
Yes	119 (29.7%)	8998 (50.3%)	
No/Unknown	281 (70.3%)	8880 (49.7%)	
Chemotherapy			P<0.01
Yes	366 (91.5%)	14506 (81.1%)	
No/Unknown	34 (8.5%)	3372 (18.9%)	
Bone metastases			P<0.01
Yes	219 (54.8%)	498 (2.8%)	
No	181 (45.2%)	17380 (97.2%)	
Lung metastases			P<0.01
Yes	82 (20.5%)	164 (0.9%)	
No	318 (79.5%)	17714 (99.1%)	
Brain metastases			P<0.01
Yes	33 (8.3%)	34 (0.2%)	
No	367 (91.7%)	17844 (99.8%)	

### Independent risk factor for YBCLM

**Figure 1:**
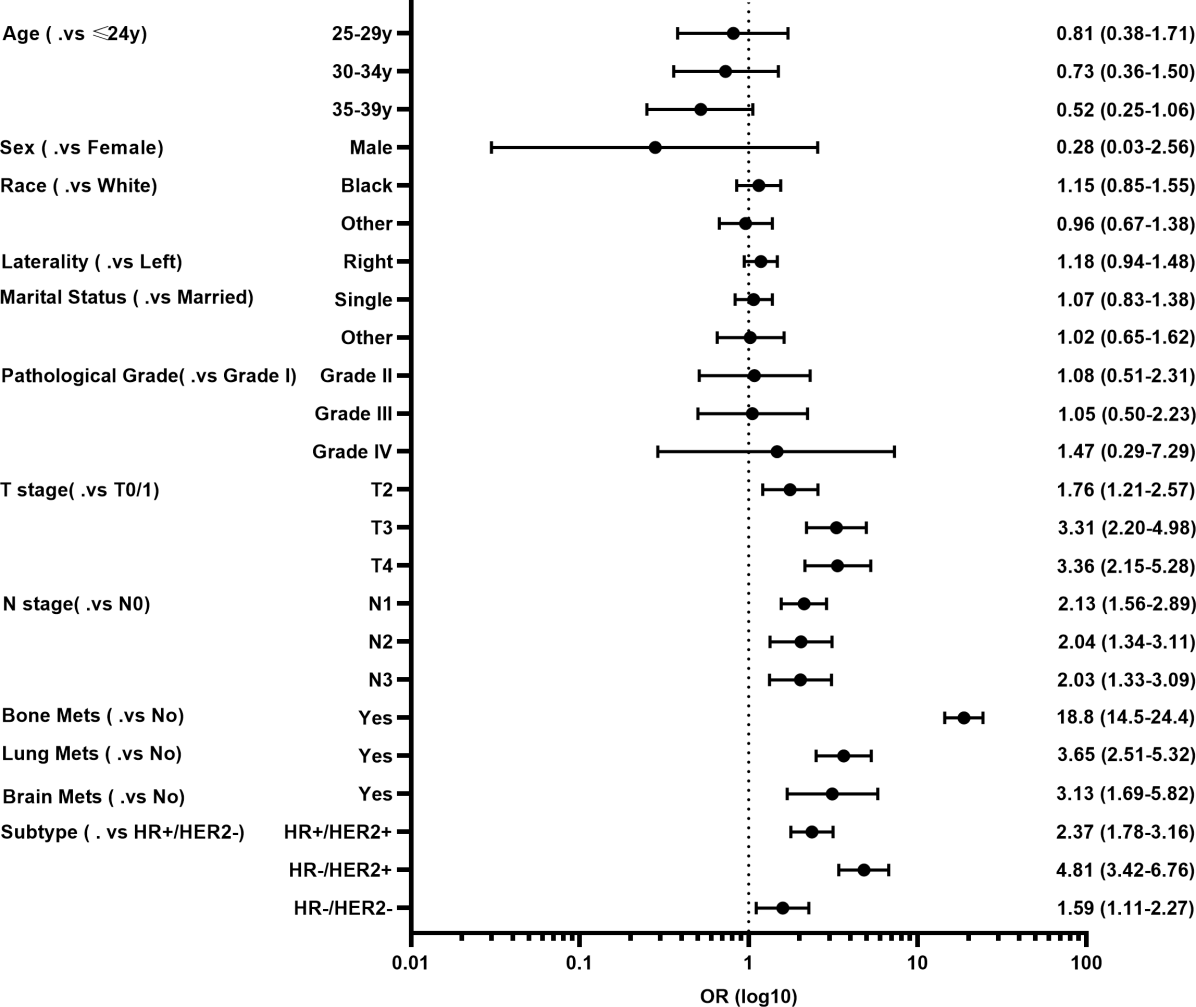
Forest plot of risk factors for developing liver metastases (LM) in young patients with breast cancer (YBC).

Next, the multivariate logistic regression analysis was performed to identify independent factors for predicting LM in YBC. As shown in **Figure 1**, T and N stages, molecular subtypes, and bone, lung, and brain metastases were independent risk factors. Among these factors, YBC with bone metastases had the highest possibility of developing LM (OR=18.8, 95% CI 14.5–24.4). As for the molecular subtypes, compared with YBC with HR+/HER2-, those with HR-/HER2+ had the highest risk of LM (OR=4.81, 95% CI 3.42–6.76), followed by HR+/HER2+ and triple-negative BC.

### Establishment of a diagnostic nomogram for YBCLM

**Figure 2:**
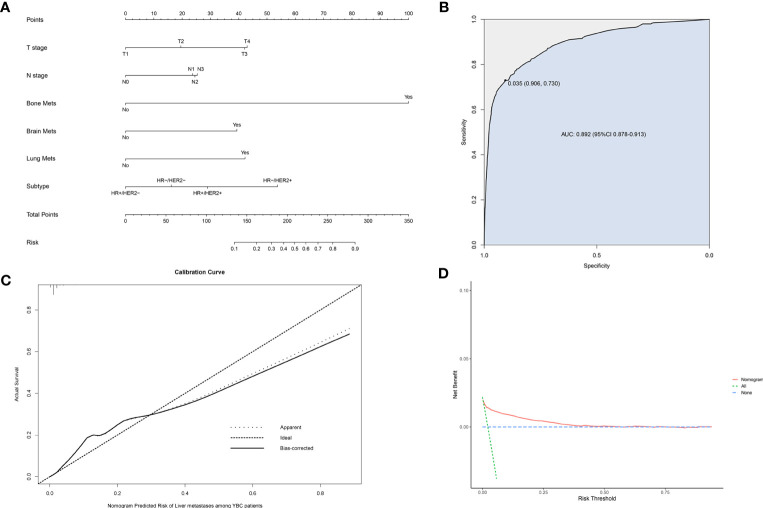
Nomogram to assess the risk of liver metastases (LM) in young patients with breast cancer (YBC) **(A)**. Receiver operating characteristic (ROC) analysis **(B)**, calibration curve **(C)**, and decision curve analysis (DCA) **(D)** are used to assess the performance of the established nomogram.

A risk prediction nomogram model for YBC developing LM was established on the basis of the independent predictive factors obtained from the multivariate logistic regression analysis. The C-index for this diagnostic nomogram was 0.895 (95% CI 0.877–0.913) (**Figure 2A**). Consistent with the results of the multivariate logistic analysis, bone metastases contributed the most risk of LM developing, followed by the molecular subtypes of HR-/HER2+, lung metastases, etc. The ROC analysis revealed that the area under the curve (AUC) value of this risk nomogram is 0.892 (95% CI 0.878–0.913), indicating that this model had excellent discriminatory power (**Figure 2B**). The calibration curve also showed that the observed results were consistent with the predicted results (**Figure 2C**). The DCA showed that the risk nomogram model is effective in clinical practice (**Figure 2D**).

### Differences in characteristics and survival between YBCLM and OBCLM

**Figure 3:**
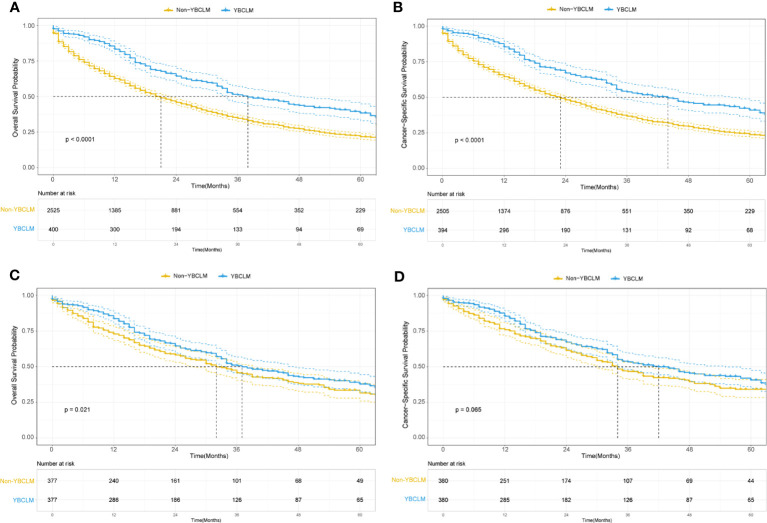
Kaplan–Meier curve of overall survival (OS) and cancer-specific survival (CSS) in young breast cancer with liver metastases (YBCLM) and non-YBCLM in the unmatched **(A, B)** and matched **(C, D)** cohorts after propensity score matching (PSM).

We also identified 2,525 OBCLM from the SEER database diagnosed between 2010 and 2019. As shown in **Table 2**, significant differences were observed in race, marital status, lung metastases, and the use of surgery and chemotherapy. YBCLM tended to be non-White, without lung metastases, and more likely to undergo surgery and chemotherapy. Survival analysis showed that YBCLM had better OS (mOS: 38 vs 21 m) and CSS (mCSS: 44 vs 23 m) than OBCLM in the unmatched cohort (P<0.01 for both) (**Figures 3A, B**). We then compared the differences in OS and CSS in the matched cohort after PSM analysis, balancing confounding factors (1:1). No significant differences were observed in any of the characteristics between YBCLM and OBCLM cases in matched cohort for OS and CSS (**Supplementary Tables 1**, **2**). In the matched cohort, the survival analysis showed that YBCLM had significantly better OS (P=0.021) than OBCLM, but not CSS (P=0.065) (**Figures 3C, D**).

**Table 2:** The demographic and clinicopathological characteristics of YBC patients with LM and OBC patients with LM.

Characteristics	YBC patients with LM (N=400)	OBC patients with LM (N=2525)	P Value
Sex			
Female	399	2509	P=0.56
Male	1	16	
Race			
White	266	1854	P<0.01
Black	91	417	
Other	43	254	
Laterality			
Left	192	1283	P=0.34
Right	208	1236	
Bilateral	0	6	
Marital status			
Married	216	1213	P<0.01
Single	159	583	
Others	25	729	
Grade			
I	9	76	P=0.33
II	125	882	
III	263	1554	
IV	3	13	
AJCC-T			
T0-1	39	280	P<0.01
T2	145	882	
T3	112	463	
T4	104	900	
AJCC-N			
N0	68	487	0.69
N1	219	1320	
N2	51	335	
N3	62	383	
Subtype			
Luminal A	132	1007	0.04
Luminal B	119	647	
HER2	84	445	
Triple-negative	65	426	
Surgery			
BCS	37	214	P<0.01
Mastectomy	101	435	
No surgery	262	1876	
Radiation			0.11
Yes	119	652	
No/Unknown	281	1873	
Chemotherapy			P<0.01
Yes	366	1832	
No/Unknown	34	693	
Bone metastases			0.35
Yes	219	1449	
No	181	1076	
Lung metastases			P<0.01
Yes	82	903	
No	318	1622	
Brain metastases			1.00
Yes	33	210	
No	367	2315	

### Independent prognostic factor for OS and CSS in YBCLM

The multivariate Cox regression analysis was used to determine significant independent prognostic factors. As shown in **Table 3**, the multivariate Cox analysis demonstrated that molecular subtypes, use of surgery, and presence of bone, lung, and brain metastases were independently associated with OS and CSS (P<0.05 for all). Chemotherapy was an independent prognostic factor for OS, whereas marital status and T stage were independent prognostic factors for CSS (P<0.05 for all).

**Table 3:** Multivariate Cox regression analysis for OS and CSS among YBC patients with LM.

Characteristics	OS		CSS	
	HR with 95%CI	P Value	HR with 95%CI	P Value
Age				
<30yr	Reference		Reference	
≥30yr	0.83 (0.64-1.42)	0.96	1.00 (0.66-1.54)	0.98
Race				
White	Reference		Reference	
Black	1.12 (0.79-1.59)	0.53	1.08 (0.75-1.56)	0.67
Other	0.96 (0.57-1.60)	0.86	0.75 (0.41-1.36)	0.34
Laterality				
Left	Reference		Reference	
Right	0.82 (0.61-1.10)	0.19	0.78 (0.57-1.07)	0.12
Marital status				
Married	Reference		Reference	
Single	1.38 (0.99-1.91)	0.06	1.45 (1.03-2.03)	0.03
Others	1.14 (0.61-2.11)	0.68	1.14 (0.60-2.18)	0.69
Grade				
I	Reference		Reference	
II	0.54 (0.20-1.46)	0.23	0.52 (0.19-1.42)	0.20
III	0.78 (0.29-2.09)	0.63	0.78 (0.29-2.10))	0.62
IV	1.61 (0.27-9.65)	0.60	1.84 (0.30-11.2)	0.51
AJCC-T				
T0/1	Reference		Reference	
T2	1.18 (0.63-2.20)	0.61	1.29 (0.65-2.54)	0.46
T3	1.20 (0.63-2.26)	0.58	1.34 (0.67-2.67)	0.41
T4	1.82 (0.96-3.44)	0.07	2.02 (1.01-4.03)	0.04
AJCC-N				
N0	Reference		Reference	
N1	0.91 (0.59-1.41)	0.66	0.90 (0.57-1.40)	0.63
N2	1.46 (0.85-2.51)	0.17	1.37 (0.77-2.43)	0.28
N3	1.16 (0.68-1.97)	0.58	1.05 (0.60-1.84)	0.86
Subtype				
Luminal A	Reference		Reference	
Luminal B	0.46 (0.31-0.68)	<0.01	0.40 (0.26-0.61)	<0.01
HER2	0.63 (0.41-0.98)	0.04	0.57 (0.36-0.91)	0.02
Triple-negative	2.26 (1.45-3.53)	<0.01	2.25 (1.41-3.59)	<0.01
Surgery				
No surgery	Reference		Reference	
BCS	0.52 (0.29-0.93)	0.03	0.50 (0.27-0.94)	0.03
Mastectomy	0.61 (0.41-0.89)	0.01	0.62 (0.41-0.93)	0.02
Radiation				
No/Unknown	Reference		Reference	
Yes	1.10 (0.78-1.55)	0.59	1.13 (0.78-1.64)	0.51
Chemotherapy				
No/Unknown	Reference		Reference	
Yes	0.49 (0.29-0.82)	<0.01	0.68 (0.38-1.23)	0.20
Bone metastases				
No/Unknown	Reference		Reference	
Yes	1.44 (1.05-1.97)	0.03	1.53 (1.09-2.14)	0.01
Lung metastases				
No/Unknown	Reference		Reference	
Yes	1.54 (1.10-2.17)	0.01	1.47 (1.03-2.11)	0.04
Brain metastases				
No/Unknown	Reference		Reference	
Yes	1.69 (1.01-2.84)	0.04	2.00 (1.17-3.41))	0.01

### Establishment of OS- and CSS-specific nomograms for YBCLM

**Figure 4:**
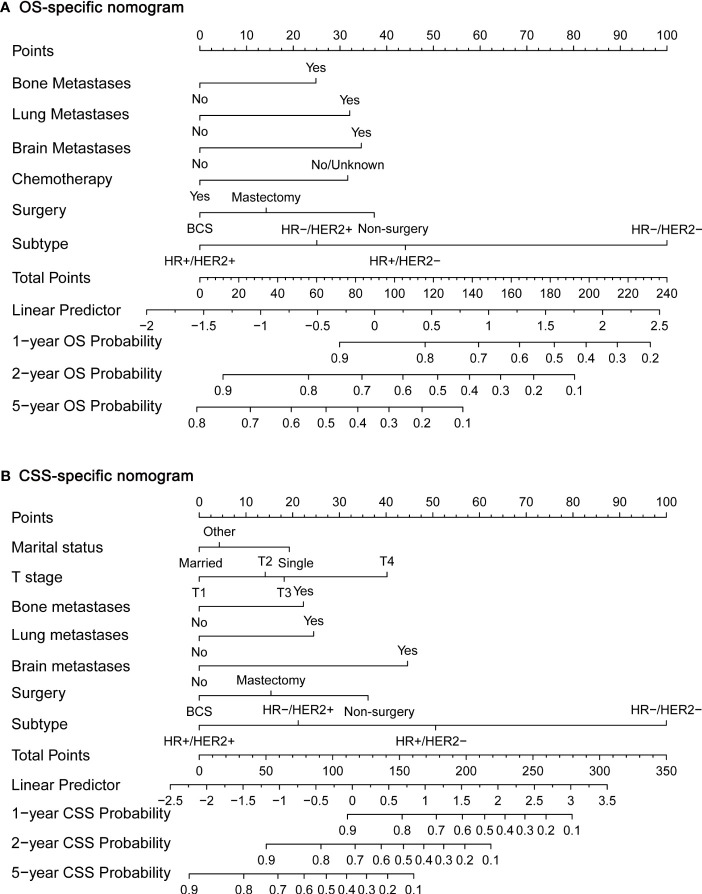
Nomograms for predicting 1-, 2-, and 5-year overall survival (OS) **(A)** and cancer-specific survival (CSS) **(B)**.

**Figure 5:**
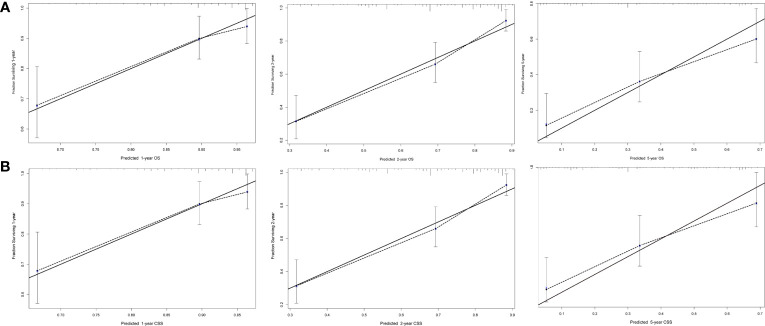
Calibration curves for predicting overall survival (OS) **(A)** and cancer-specific survival (CSS) **(B)** at 1, 2, and 5 years.

**Figure 6:**
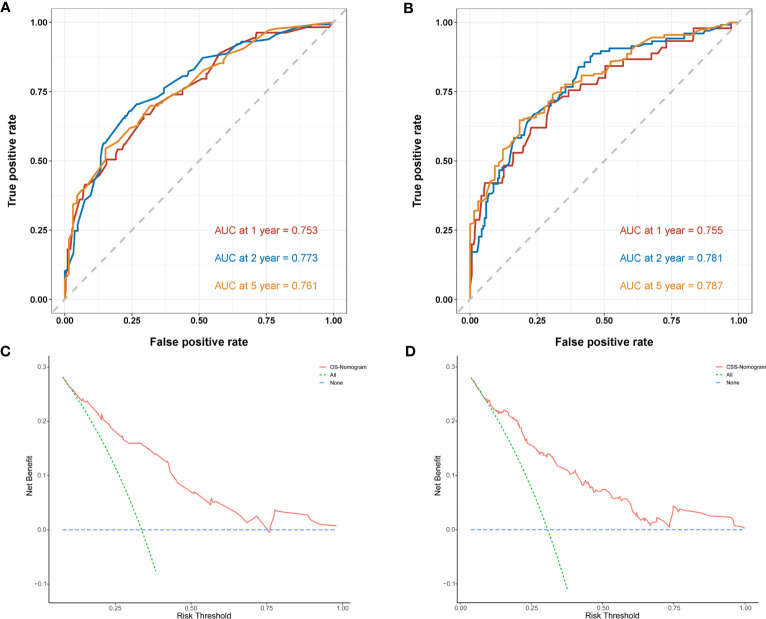
Time-dependent receiver operating characteristic (ROC) curves of the overall survival (OS)-specific nomogram **(A)** and cancer-specific survival (CSS)-specific nomogram **(B)** for 1-, 2-, and 5-year predictions. Decision curve analysis (DCA) of the overall survival (OS)-specific nomogram **(C)** and cancer-specific survival (CSS)-specific nomogram **(D)**.

Next, we established OS- and CSS-specific prognostic nomogram models using the abovementioned independent prognostic factors to predict the survival of YBCLM. As shown in **Figure 4A**, molecular subtypes had the most influence on the OS-specific nomogram, followed by the use of surgery and chemotherapy. Regarding the CSS-specific nomogram, molecular subtypes were also the leading prognostic factor, followed by brain metastases and the use of surgery (**Figure 4B**). Among these factors, the triple-negative subtype accounted for the highest risk of death due to BC or other reasons. The C-indices for the OS- and CSS-specific nomograms were 0.728 (0.69–0.766) and 0.74 (0.696–0.778), respectively, suggesting that both prediction models had a good discrimination power. The calibration curve of the OS- and CSS-specific nomograms demonstrated that the predicted probability was consistent with the actual probability for OS and CSS predictions, suggesting an accurate predictive effect of the established nomogram (**Figure 5**). The ROC analysis showed that the AUC values of the OS-specific nomogram for 1-, 2-, and 5-year OS were 0.753, 0.773, and 0.761, respectively, and those for the CSS-specific nomogram were 0.755, 0.781, and 0.787, respectively (**Figures 6A, B**). The DCA was also conducted to assess the clinical practice value of these nomograms in predicting the survival of YBCLM. As illustrated in **Figures 6C, D**, the DCA suggested that the OS- and CSS-specific nomograms had a significant positive net benefit from the risk of death, indicating their real-world clinical practice value in predicting survival.

## Discussion

Although LM are not an uncommon metastatic pattern in BC, few studies have focused on risk and survival analyses for YBCLM. It remained unclear about the clinicopathological characteristics and survival of YBCLM patients, especially the difference between YBCLM and OBCLM. Thus, we conducted this study using the data from SEER database. To the best of our knowledge, our study is the first population-based comprehensive retrospective study focus on YBC patients with liver metastases. First, we described clinicopathological features and prognosis of YBCLM patients, and compared with YBC patients without LM, OBC patients with LM. Second, improving the survival of YBC, requires timely evaluation of the risk probability of LM in YBC and the survival probability of YBCLM during early screening, which could facilitate early prevention and clinical interventions to prolong survival. Thus, the present study also identified the risk factor for developing LM, as well as prognostic factors associated with OS and CSS among YBCLM patients. Meanwhile, the risk and prognostic nomograms for YBCLM were constructed and validated, which could help clinicians accurately predict the development of LM and survival in YBC.

The data showed that T and N stages, molecular subtypes, and bone, lung, and brain metastases were significantly associated with the risk of developing LM in YBC. T or N stage can often reflect tumor burden and depth and the extent of tumor invasion, so an advanced T or N stage representing a high risk of distant metastases, including LM, is doubtful. The bone is the most common metastatic site of BC, accounting for approximately 75% of metastatic cases, and the lung is the second most frequent metastasis site. Approximately 15–30% of metastatic BC cases can develop brain metastases, indicating an extremely short survival time (19). Bone and lung metastases often have better OS than LM (20). All these metastatic patterns can increase the risk of LM occurring (21), especially bone metastases. Our data demonstrated that bone metastases contributed the most risk of LM developing in YBC. YBC with bone metastases have more than an 18-fold risk of LM development, suggesting YBC patients with bone metastases are generally at a high risk of developing multiple distant organ metastases, especially liver metastases.

In addition, the possibility or risk of developing liver metastases varied among patients with different subtypes of breast cancer. Our data also confirmed that those patients with the HER2+ or triple-negative subtype had a significantly greater risk of developing LM than those with the HR+/HER2- subtype in YBC, which is consistent with previous research on the metastatic patterns of different BC subtypes (22–24). Mechanistically, previous studies have demonstrated the potential association of cytokines, chemokine receptors, and neutrophil extracellular traps with LM among different subtypes of BC (25–27). For example, previous evidence has demonstrated that HER2 could upregulate the expression of chemokine receptor-4 (CXCR4), thereby promoting LM via the CXCL12/CXCR4 signaling pathway (28), and overexpression of fibroblast growth factor homologous factor could induce the formation and occurrence of LM in triple-negative BC (29). In general, these findings underscore that BC molecular subtypes and properties may be associated with the correlation between specific features of the tumor and metastases to specific sites, resulting in molecular subtype-based LM of BC. Due to the design of retrospective study, we are not able to evaluate the association between molecular subtypes and liver metastases of YBC patients mechanistically.

It has been known that novel treatment options and different metastatic sites have profoundly changed the prognostic value of molecular subtypes in patients with BC. Historically, HR+/HER2- BC has been considered to have a favorable prognosis. After the clinical application of anti-HER2-targeted therapy, patients with HR+/HER2+ seemed to have the best prognosis, whereas those with triple-negative BC had the worst survival among metastatic BCs (30). Patients with HR2+/HER2- and HR-/HER2+ are considered to have similar survival rates among patients with metastatic BC (31). Our data also showed that YBCLM patients with HR-/HER2+ have a relatively better OS and CSS than those with HR+/HER2-, which is consistent with the data of a previous report (32). Previous studies have provided evidence for this discrepancy. Compared with patients with HR+/HER2- BC without visceral metastases, those with visceral metastases are less sensitive to endocrine therapy, leading to a worse prognosis (33, 34). Meanwhile, the spatial heterogeneity of distant metastases may contribute to the different responses to endocrine therapy among patients with metastatic HR+/HER2- BC (34). He et al. reported that patients with HR+/HER2- BC with LM who received fulvestrant therapy had significantly shorter median progression-free survival than those with lung metastases (33). With substantial progress in our understanding of the biology of HER+/HER2- BC, novel targeted drugs, such as CDK4/6 inhibitors, have been developed (35). A subsequent study demonstrated that patients with HR+/HER2- BC with visceral metastases could benefit from combining novel targeted therapy with endocrine therapy (36–38). Thus, endocrine therapy plus targeted therapy may be more appropriate for YBCLM with HR+/HER2-, but this should be confirmed in future trials. Except for systematic treatment, local treatments for BC with LM including surgery and radiotherapy, are most commonly used when the liver is the only site of metastasis and liver oligometastases (39). The role of liver resection and ablation in the modern treatment of BCLM remains unclear. Several case series have shown the clinical benefits of surgery or ablation in conjunction with systemic therapy (40–42). Studies have revealed the prognostic factors useful for choosing eligible patients for surgery, including single LM, estrogen-positive BC, non-triple negative tumor, and a good response to systemic treatment (43–45). Meanwhile, studies opposing liver surgery argue that it might delay or even interrupt systemic treatment; however, these studies lack a high level of evidence and have a low sample size. Sunden et al. conducted a nationwide registry-based study demonstrating that surgery for BCLM is safe and has a survival benefit in one in five patients with BCLM without extrahepatic spread (45). Consistent with the findings of this study, our data also indicated a clinical benefit of surgery for YBCLM, regardless of OS or CSS. Although there was no further detailed information on the YBCLM who underwent surgery in our study, all this evidence suggests that surgery may play a vital role in these patients, especially those with advantageous factors for selecting surgery. Owing to the nature of retrospective studies, prospective trials that avoid selection bias are warranted to elucidate the accurate potential of surgery in patients with BCLM.

Obviously, our study also had several potential limitations, similar to other SEER database-based population studies, that cannot be neglected (15, 46). First, the nomogram was based only on retrospective studies without any external validation cohorts, which may limit the accuracy and reliability of these results. Second, because of the retrospective nature of the studies, selection bias is unavoidable. Third, the SEER database only provides information regarding distant metastases from 2010, with the omission of previous cases, which also limits the sample size. Fourth, some potentially crucial features, including the number and location of LM, information on endocrine and targeted therapies, sequence of distant organ metastases and liver function, are unavailable in the SEER database, which influences the reliability of the outcomes. Despite the established nomogram showing excellent predictive performance, more YBCLM cases from a real-world cohort are required to confirm our results.

In conclusion, we identified the risk and prognostic factors of BC with LM in young patients using the data from the SEER database and constructed nomograms to predict the diagnosis and prognosis of YBCLM, which has excellent predictive performance and clinical utility. These nomogram models can help physicians execute tailored risk assessments, clinical decision-making, and follow-up plans. These results are also essential for the management of YBC patients and for further prospective studies on this disease.

## Data availability statement

The raw data supporting the conclusions of this article will be made available by the authors, without undue reservation.

## Ethics statement

Ethical review and approval was not required for the study on human participants in accordance with the local legislation and institutional requirements. Written informed consent for participation was not required for this study in accordance with the national legislation and the institutional requirements.

## Author contributions

C-CP: Study design, Date collects, Data analysis, Writing- Original draft preparation. LY: Date collect, Data analysis, Writing- Original draft preparation. J-MY: Study design, Supervision, writing- Reviewing and editing. All authors contributed to the article and approved the submitted version.

## References

[1] Sung H Ferlay J Siegel RL Laversanne M Soerjomataram I Jemal A. Global cancer statistics 2020: GLOBOCAN estimates of incidence and mortality worldwide for 36 cancers in 185 countries. CA Cancer J Clin (2021) 71(3):209–49. doi: 10.3322/caac.21660 33538338

[2] Kataoka A Iwamoto T Tokunaga E Tomotaki A Kumamaru H Miyata H. Young adult breast cancer patients have a poor prognosis independent of prognostic clinicopathological factors: a study from the Japanese breast cancer registry. Breast Cancer Res Treat (2016) 160(1):163–72. doi: 10.1007/s10549-016-3984-8 PMC5050233 27647460

[3] Walsh SM Zabor EC Flynn J Stempel M Morrow M Gemignani ML. Breast cancer in young black women. Br J Surg (2020) 107(6):677–86. doi: 10.1002/bjs.11401 PMC7422000 31981221

[4] Huang X Luo Z Liang W Xie G Lang X Gou J. Survival nomogram for young breast cancer patients based on the SEER database and an external validation cohort. Ann Surg Oncol (2022) 29(9):5772–81. doi: 10.1245/s10434-022-11911-8 PMC9356966 35661275

[5] Paluch-Shimon S Cardoso F Partridge AH Abulkhair O Azim HA Jr. Bianchi-Micheli G. ESO-ESMO 4th international consensus guidelines for breast cancer in young women (BCY4). Ann Oncol (2020) 31(6):674–96. doi: 10.1016/j.annonc.2020.03.284 32199930

[6] Johnson RH Anders CK Litton JK Ruddy KJ Bleyer A. Breast cancer in adolescents and young adults. Pediatr Blood Cancer (2018) 65(12):e27397. doi: 10.1002/pbc.27397 30156052 PMC6192832

[7] Villarreal-Garza C Platas A Miaja M Fonseca A Mesa-Chavez F Garcia-Garcia M. Young women with breast cancer in Mexico: results of the pilot phase of the joven & fuerte prospective cohort. JCO Glob Oncol (2020) 6:395–406. doi: 10.1200/JGO.19.00264 32142405 PMC7113130

[8] Tung N Battelli C Allen B Kaldate R Bhatnagar S Bowles K. Frequency of mutations in individuals with breast cancer referred for BRCA1 and BRCA2 testing using next-generation sequencing with a 25-gene panel. Cancer (2015) 121(1):25–33. doi: 10.1002/cncr.29010 25186627

[9] Murphy BL Day CN Hoskin TL Habermann EB Boughey JC. Adolescents and young adults with breast cancer have more aggressive disease and treatment than patients in their forties. Ann Surg Oncol (2019) 26(12):3920–30. doi: 10.1245/s10434-019-07653-9 31376035

[10] Tzikas AK Nemes S Linderholm BK. A comparison between young and old patients with triple-negative breast cancer: biology, survival and metastatic patterns. Breast Cancer Res Treat (2020) 182(3):643–54. doi: 10.1007/s10549-020-05727-x PMC7320950 32524352

[11] Cancer Genome Atlas N. Comprehensive molecular portraits of human breast tumours. Nature (2012) 490(7418):61–70. doi: 10.1038/nature11412 23000897 PMC3465532

[12] Valastyan S Weinberg RA. Tumor metastasis: molecular insights and evolving paradigms. Cell (2011) 147(2):275–92. doi: 10.1016/j.cell.2011.09.024 PMC3261217 22000009

[13] Allemani C Matsuda T Di Carlo V Harewood R Matz M Niksic M. Global surveillance of trends in cancer survival 2000-14 (CONCORD-3): analysis of individual records for 37 513 025 patients diagnosed with one of 18 cancers from 322 population-based registries in 71 countries. Lancet (2018) 391(10125):1023–75. doi: 10.1016/S0140-6736(17)33326-3 PMC5879496 29395269

[14] Li Q Cai G Li D Wang Y Zhuo C Cai S. Better long-term survival in young patients with non-metastatic colorectal cancer after surgery, an analysis of 69,835 patients in SEER database. PloS One (2014) 9(4):e93756. doi: 10.1371/journal.pone.0093756 24699879 PMC3974782

[15] Yao ZX Tu JH Zhou B Huang Y Liu YL Xue XF. Risk factors and survival prediction of pancreatic cancer with lung metastases: a population-based study. Front Oncol (2022) 12:952531. doi: 10.3389/fonc.2022.952531 36212473 PMC9533144

[16] Kane LT Fang T Galetta MS Goyal DKC Nicholson KJ Kepler CK. Propensity score matching: a statistical method. Clin Spine Surg (2020) 33(3):120–2. doi: 10.1097/BSD.0000000000000932 31913173

[17] Longato E Vettoretti M Di Camillo B. A practical perspective on the concordance index for the evaluation and selection of prognostic time-to-event models. J BioMed Inform (2020) 108:103496. doi: 10.1016/j.jbi.2020.103496 32652236

[18] Van Calster B Wynants L Verbeek JFM Verbakel JY Christodoulou E Vickers AJ. Reporting and interpreting decision curve analysis: a guide for investigators. Eur Urol (2018) 74(6):796–804. doi: 10.1016/j.eururo.2018.08.038 30241973 PMC6261531

[19] Liang Y Zhang H Song X Yang Q. Metastatic heterogeneity of breast cancer: molecular mechanism and potential therapeutic targets. Semin Cancer Biol (2020) 60:14–27. doi: 10.1016/j.semcancer.2019.08.012 31421262

[20] Pentheroudakis G Fountzilas G Bafaloukos D Koutsoukou V Pectasides D Skarlos D. Metastatic breast cancer with liver metastases: a registry analysis of clinicopathologic, management and outcome characteristics of 500 women. Breast Cancer Res Treat (2006) 97(3):237–44. doi: 10.1007/s10549-005-9117-4 16322882

[21] Ji L Cheng L Zhu X Gao Y Fan L Wang Z. Risk and prognostic factors of breast cancer with liver metastases. BMC Cancer (2021) 21(1):238. doi: 10.1186/s12885-021-07968-5 33676449 PMC7937288

[22] Sihto H Lundin J Lundin M Lehtimaki T Ristimaki A Holli K. Breast cancer biological subtypes and protein expression predict for the preferential distant metastasis sites: a nationwide cohort study. Breast Cancer Res (2011) 13(5):R87. doi: 10.1186/bcr2944 21914172 PMC3262199

[23] Purushotham A Shamil E Cariati M Agbaje O Muhidin A Gillett C. Age at diagnosis and distant metastasis in breast cancer–a surprising inverse relationship. Eur J Cancer (2014) 50(10):1697–705. doi: 10.1016/j.ejca.2014.04.002 24768572

[24] Soni A Ren Z Hameed O Chanda D Morgan CJ Siegal GP. Breast cancer subtypes predispose the site of distant metastases. Am J Clin Pathol (2015) 143(4):471–8. doi: 10.1309/AJCPYO5FSV3UPEXS 25779997

[25] Chen W Hoffmann AD Liu H Liu X. Organotropism: new insights into molecular mechanisms of breast cancer metastasis. NPJ Precis Oncol (2018) 2(1):4. doi: 10.1038/s41698-018-0047-0 29872722 PMC5871901

[26] Yang L Liu Q Zhang X Liu X Zhou B Chen J. DNA Of neutrophil extracellular traps promotes cancer metastasis via CCDC25. Nature (2020) 583(7814):133–8. doi: 10.1038/s41586-020-2394-6 32528174

[27] Zlotnik A Burkhardt AM Homey B. Homeostatic chemokine receptors and organ-specific metastasis. Nat Rev Immunol (2011) 11(9):597–606. doi: 10.1038/nri3049 21866172

[28] Li YM Pan Y Wei Y Cheng X Zhou BP Tan M. Upregulation of CXCR4 is essential for HER2-mediated tumor metastasis. Cancer Cell (2004) 6(5):459–69. doi: 10.1016/j.ccr.2004.09.027 15542430

[29] Johnstone CN Pattison AD Harrison PF Powell DR Lock P Ernst M. FGF13 promotes metastasis of triple-negative breast cancer. Int J Cancer (2020) 147(1):230–43. doi: 10.1002/ijc.32874 31957002

[30] Leone JP Leone J Zwenger AO Vallejo CT Leone BA. Prognostic significance of tumor subtypes in women with breast cancer according to stage: a population-based study. Am J Clin Oncol (2019) 42(7):588–95. doi: 10.1097/COC.0000000000000563 31166208

[31] Gong Y Liu YR Ji P Hu X Shao ZM. Impact of molecular subtypes on metastatic breast cancer patients: a SEER population-based study. Sci Rep (2017) 7:45411. doi: 10.1038/srep45411 28345619 PMC5366953

[32] Arciero CA Guo Y Jiang R Behera M O’Regan R Peng L. ER(+)/HER2(+) breast cancer has different metastatic patterns and better survival than ER(-)/HER2(+) breast cancer. Clin Breast Cancer (2019) 19(4):236–45. doi: 10.1016/j.clbc.2019.02.001 30846407

[33] He M Li JJ Zuo WJ Ji L Jiang YZ Hu XC. Metastatic breast cancer patients with lung or liver metastases should be distinguished before being treated with fulvestrant. Cancer Med (2019) 8(14):6212–20. doi: 10.1002/cam4.2453 PMC6797565 31373147

[34] Robertson JFR Bondarenko IM Trishkina E Dvorkin M Panasci L Manikhas A. Fulvestrant 500 mg versus anastrozole 1 mg for hormone receptor-positive advanced breast cancer (FALCON): an international, randomised, double-blind, phase 3 trial. Lancet (2016) 388(10063):2997–3005. doi: 10.1016/S0140-6736(16)32389-3 27908454

[35] Turner NC Neven P Loibl S Andre F. Advances in the treatment of advanced oestrogen-receptor-positive breast cancer. Lancet (2017) 389(10087):2403–14. doi: 10.1016/S0140-6736(16)32419-9 27939057

[36] Andre F Ciruelos E Rubovszky G Campone M Loibl S Rugo HS. Alpelisib for PIK3CA-mutated, hormone receptor-positive advanced breast cancer. N Engl J Med (2019) 380(20):1929–40. doi: 10.1056/NEJMoa1813904 31091374

[37] Jiang Z Li W Hu X Zhang Q Sun T Cui S. Tucidinostat plus exemestane for postmenopausal patients with advanced, hormone receptor-positive breast cancer (ACE): a randomised, double-blind, placebo-controlled, phase 3 trial. Lancet Oncol (2019) 20(6):806–15. doi: 10.1016/S1470-2045(19)30164-0 31036468

[38] Turner NC Finn RS Martin M Im SA DeMichele A Ettl J. Clinical considerations of the role of palbociclib in the management of advanced breast cancer patients with and without visceral metastases. Ann Oncol (2018) 29(3):669–80. doi: 10.1093/annonc/mdx797 PMC5888946 29342248

[39] Zuo Q Park NH Lee JK Madak Erdogan Z. Liver metastatic breast cancer: epidemiology, dietary interventions, and related metabolism. Nutrients (2022) 14(12):2376. doi: 10.3390/nu14122376 35745105 PMC9228756

[40] Bacalbasa N Dima SO Purtan-Purnichescu R Herlea V Popescu I. Role of surgical treatment in breast cancer liver metastases: a single center experience. Anticancer Res (2014) 34(10):5563–8. 25275056

[41] Charalampoudis P Mantas D Sotiropoulos GC Dimitroulis D Kouraklis G Markopoulos C. Surgery for liver metastases from breast cancer. Future Oncol (2015) 11(10):1519–30. doi: 10.2217/fon.15.43 25963429

[42] Vertriest C Berardi G Tomassini F Vanden Broucke R Depypere H Cocquyt V. Resection of single metachronous liver metastases from breast cancer stage I-II yield excellent overall and disease-free survival. single center experience and review of the literature. Dig Surg (2015) 32(1):52–9. doi: 10.1159/000375132 25675843

[43] Abbott DE Brouquet A Mittendorf EA Andreou A Meric-Bernstam F Valero V. Resection of liver metastases from breast cancer: estrogen receptor status and response to chemotherapy before metastasectomy define outcome. Surgery (2012) 151(5):710–6. doi: 10.1016/j.surg.2011.12.017 PMC3628698 22285778

[44] Hoffmann K Franz C Hinz U Schirmacher P Herfarth C Eichbaum M. Liver resection for multimodal treatment of breast cancer metastases: identification of prognostic factors. Ann Surg Oncol (2010) 17(6):1546–54. doi: 10.1245/s10434-010-0931-5 20143267

[45] Sunden M Hermansson C Taflin H Andersson A Sund M Hemmingsson O. Surgical treatment of breast cancer liver metastases - a nationwide registry-based case control study. Eur J Surg Oncol (2020) 46(6):1006–12. doi: 10.1016/j.ejso.2020.02.008 32098734

[46] Zhang W Ji L Wang X Zhu S Luo J Zhang Y. Nomogram predicts risk and prognostic factors for bone metastasis of pancreatic cancer: a population-based analysis. Front Endocrinol (Lausanne) (2021) 12:752176. doi: 10.3389/fendo.2021.752176 35356148 PMC8959409

