# Supplementary material for: Risk factors and survival prediction of young breast cancer patients with liver metastases: a population-based study
Source: Front Endocrinol (Lausanne). 2023 Jun 23;14:1158759. doi: 10.3389/fendo.2023.1158759 (PMC10328090; doi:10.3389/fendo.2023.1158759)
Supplement: Supplementary file 1 [file Table_1.docx]

**Supplementary Table 1**: The demographic and clinicopathological characteristics of YBC patients with LM and OBC patients with LM in matched cohort for OS analysis

| Characteristics | YBC patients with LM (N=377） | OBC patients with LM (N=377) | P |
| --- | --- | --- | --- |
| Sex |  |  |  |
| Female | 376 | 377 | P=1.00 |
| Male | 1 | 0 |  |
| Race |  |  |  |
| White | 255 | 245 | P=0.68 |
| Black | 81 | 91 |  |
| Other | 41 | 41 |  |
| Laterality |  |  |  |
| Left | 173 | 183 | P=0.51 |
| Right | 194 | 184 |  |
| Bilateral | 0 | 0 |  |
| Marital status |  |  |  |
| Married | 211 | 206 | P=0.40 |
| Single | 141 | 136 |  |
| Others | 25 | 35 |  |
| Grade |  |  |  |
| I | 9 | 9 | P=0.47 |
| II | 122 | 107 |  |
| III | 244 | 256 |  |
| IV | 2 | 5 |  |
| AJCC-T |  |  |  |
| T0-1 | 39 | 26 | P=0.30 |
| T2 | 141 | 140 |  |
| T3 | 93 | 108 |  |
| T4 | 104 | 103 |  |
| AJCC-N |  |  |  |
| N0 | 64 | 58 | P=0.89 |
| N1 | 205 | 215 |  |
| N2 | 49 | 47 |  |
| N3 | 59 | 57 |  |
| Subtype |  |  |  |
| Luminal A | 128 | 120 | P=0.66 |
| Luminal B | 113 | 111 |  |
| HER2 | 76 | 73 |  |
| Triple-negative | 60 | 73 |  |
| Surgery |  |  |  |
| BCS | 36 | 40 | P=0.77 |
| Mastectomy | 90 | 83 |  |
| No surgery | 251 | 254 |  |
| Radiation |  |  | P=0.41 |
| Yes | 105 | 94 |  |
| No/Unknown | 272 | 283 |  |
| Chemotherapy |  |  | P=0.70 |
| Yes | 343 | 347 |  |
| No/Unknown | 34 | 30 |  |
| Bone metastases |  |  | P=0.42 |
| Yes | 207 | 219 |  |
| No | 170 | 158 |  |
| Lung metastases |  |  | P=0.93 |
| Yes | 82 | 80 |  |
| No | 295 | 297 |  |
| Brain metastases |  |  | P=0.79 |
| Yes | 29 | 32 |  |
| No | 348 | 345 |  |

**Supplementary Table 2**: The demographic and clinicopathological characteristics of YBC patients with LM and OBC patients with LM in matched cohort for CSS analysis

| Characteristics | YBC patients with LM (N=380） | OBC patients with LM (N=380) | P |
| --- | --- | --- | --- |
| Sex |  |  |  |
| Female | 379 | 380 | P=1.00 |
| Male | 1 | 0 |  |
| Race |  |  |  |
| White | 258 | 260 | P=0.99 |
| Black | 40 | 39 |  |
| Other | 82 | 81 |  |
| Laterality |  |  |  |
| Left | 185 | 174 | P=047 |
| Right | 195 | 206 |  |
| Bilateral | 0 | 0 |  |
| Marital status |  |  |  |
| Married | 212 | 209 | P=0.91 |
| Single | 104 | 144 |  |
| Others | 24 | 27 |  |
| Grade |  |  |  |
| I | 9 | 8 | P=0.78 |
| II | 121 | 121 |  |
| III | 247 | 250 |  |
| IV | 3 | 1 |  |
| AJCC-T |  |  |  |
| T0-1 | 38 | 32 | P=0.78 |
| T2 | 143 | 155 |  |
| T3 | 101 | 97 |  |
| T4 | 98 | 96 |  |
| AJCC-N |  |  |  |
| N0 | 65 | 57 | P=0.33 |
| N1 | 209 | 222 |  |
| N2 | 50 | 37 |  |
| N3 | 56 | 64 |  |
| Subtype |  |  |  |
| Luminal A | 127 | 125 | P=0.99 |
| Luminal B | 112 | 112 |  |
| HER2 | 77 | 81 |  |
| Triple-negative | 64 | 62 |  |
| Surgery |  |  |  |
| BCS | 35 | 31 | P=0.56 |
| Mastectomy | 92 | 82 |  |
| No surgery | 253 | 267 |  |
| Radiation |  |  | P=0.94 |
| Yes | 104 | 102 |  |
| No/Unknown | 276 | 278 |  |
| Chemotherapy |  |  | P=1.00 |
| Yes | 348 | 348 |  |
| No/Unknown | 32 | 32 |  |
| Bone metastases |  |  | P=0.56 |
| Yes | 210 | 219 |  |
| No | 170 | 161 |  |
| Lung metastases |  |  | P=0.47 |
| Yes | 71 | 80 |  |
| No | 309 | 300 |  |
| Brain metastases |  |  | P=0.90 |
| Yes | 31 | 33 |  |
| No | 349 | 347 |  |
